# Awareness of moderate-to-vigorous physical activity: can information on guidelines prevent overestimation?

**DOI:** 10.1186/s12889-015-1705-6

**Published:** 2015-04-17

**Authors:** Emily CL Knox, Ian M Taylor, Stuart JH Biddle, Lauren B Sherar

**Affiliations:** British Heart Foundation National Centre for Physical Activity and Health, Loughborough University, Loughborough, LE113TU UK; School of Sport, Exercise and Health Sciences, Loughborough University, Loughborough, LE113TU UK; The NIHR Leicester- Loughborough Diet, Lifestyle and Physical Activity Biomedical Research Unit, Leicester and Loughborough, Leicestershire, LE54PW and LE113TU UK; Now at Institute of Sport, Exercise & Active Living, Victoria University, Melbourne, 8001 Australia

**Keywords:** Physical activity, Mass-media campaigns, Guidelines, Knowledge, Awareness

## Abstract

**Background:**

Mass-media campaigns such as Change4Life use messaging to promote physical activity guidelines. Raising knowledge of MVPA guidelines within UK adults is a main goal of current mass media campaigns aimed at increasing engagement in MVPA. As this may help to inform accurate perceptions of adults’ own MVPA level it is an important area of investigation. Subjective norms, health status and normal walking intensity may also influence adult’s awareness of their own MVPA behaviour. The aim of this study was to examine the hypothesis that greater knowledge of MVPA guidelines, supportive subjective norms, lower self-reported health status and intensity of typical walking pace are associated with accurate awareness of MVPA engagement within a sample of UK adults.

**Methods:**

A cross-sectional study of UK adults was conducted. UK adults who subscribed to the National Academic Mailing List Service (JISCMail) were sent an invitation to complete an online survey. 1,724 UK adults completed the online survey which included items on minutes spent in MVPA, awareness of MVPA using constructs highlighted by the precaution adoption process model, subjective norms, knowledge of guidelines, health status and demographics.

**Results:**

The sample was 70% female, 57% aged under 45, 93% White and 69% in full-time employment. 62% reported their health to be above average, while 62% demonstrated accurate awareness of their own physical activity level, only 18% correctly reported the MVPA guidelines and 51% reported high subjective norms towards MVPA. Logistic regression analyses identified high subjective norms (OR = 1.84, CI: 1.29, 2.63, *p* = .001), average or below average health status (OR = .71, CI: .53 .97, *p* = .001), and a self-reported regular walking pace of moderate-to-vigorous (OR = 1.31, CI: 1.05, 1.63, *p* = .02) to be associated with accurate MVPA awareness. Knowledge of MVPA guidelines was not associated with MVPA awareness.

**Conclusions:**

Mass media campaigns, such as Change4Life, inform the general public of MVPA guidelines. Campaign messages may be more influential targeting subjective norms instead of knowledge of guidelines, thereby raising awareness of personal MVPA behaviour amongst inactive adults and increasing motivation to engage in more MVPA.

## Background

Engagement in regular physical activity is associated with numerous health benefits including improved cardiovascular health and quality of life e.g. Glazer et al. [1] and Gill et al. [2]. Efforts have been made to align physical activity recommendations between countries (for example, Canada [3], United States [4], United Kingdom [5], Australia [6], as well as with the World Health Organization (WHO; [7]). This has resulted in the recommendation that adults engage in a minimum of 150 minutes a week of moderate-to-vigorous physical activity [MVPA]. Only ~5% of adults from the UK [8] and US [9] and 15% from Canada [10] achieve enough MVPA to meet national guidelines.

Judgements around the adequacy of an individual’s level of engagement with MVPA are informed by perceptions of how much MVPA one *should* engage in, relative to how much MVPA one *does* engage in. Inactive individuals do not always perceive themselves as being inactive. An accurate awareness that one does not engage in sufficient MVPA can increase motivation to engage in more physical activity [11,12]. Evidence suggests that many adults misjudge themselves as being more active than they are in reality, with many believing themselves to be active when in fact they do not meet MVPA guidelines [13-15]. This could be a major factor contributing to a lack of engagement in MVPA.

Physical activity campaigns around the world such as ‘Change4Life’ in the UK, ‘Fulfil a Lifetime’ in New Zealand and ‘Be Active’ in Australia, promote MVPA guidelines in order to increase knowledge of the amount of MVPA required for good health for all adults within the general population. This knowledge can be used to inform more accurate judgements of personal MVPA and motivate increased engagement. Accurate knowledge of the amount of physical activity required for health should inform more accurate judgements of whether one engages in sufficient physical activity, yet no study has previously investigated the relationship between knowledge of guidelines and awareness of physical activity behaviour. Subjective norms could be a competing influence on judgements regarding how much MVPA one should engage in and subsequently the manner in which an individual rates their own level of physical activity [16]. For instance, an individual who engages in below recommended levels of MVPA may perceive themselves to be active if their family and friends do not engage in any at all. From a health promotion standpoint it is important to know whether educating the population about the required level of MVPA is likely to influence awareness of personal levels of MVPA despite the presence of existing subjective norms.

Physical activity campaigns can also create norms through the activities promoted in their messages. For instance, campaigns often specifically promote walking as MVPA, despite evidence suggesting that walking is often performed at an insufficient intensity to meet MVPA guidelines [17-19]. This could lead to misperceptions of personal MVPA amongst those who engage in low intensity walking; however, this has yet to be empirically tested. Previous studies have also found that subjective indicators of health such as having a normal weight status can contribute towards the fallacy that one does not need to engage in physical activity and thus result in misperceptions of current MVPA engagement [15,16].

The aim of this study was to examine the hypothesis that greater knowledge of MVPA guidelines, supportive subjective norms and lower self-reported health status are associated with accurate awareness of MVPA engagement within a sample of UK adults. In addition, the intensity with which individuals typically walked was investigated as an additional predictor of accurate awareness of physical activity engagement.

## Methods

Ethical approval was granted by the ethics committee at Loughborough University. An online survey was developed using a commercially available software tool (www.surveymonkey.com). The survey was disseminated across the UK via JISC mailing lists to a convenience sample of UK adults aged 18 and above in February and March 2013. Adults who subscribed to one of 152 lists which ranged in topic from societal lists (e.g. minority ethnic groups) to occupational lists (e.g. masonry), were sent an email inviting them to complete the survey. There were 47, 950 UK subscriptions attached to these lists. A total of 2,332 participants responded to the survey which contained the following measures:

### Demographics

Participants self-reported gender, age (e.g. 16–24), ethnic background (e.g. White British), marital status (e.g. Married/Civil partnership), highest level of education (e.g. Postgraduate/Professional degree) and level of employment (e.g. full-time).

### Health status

Participants responded to the sentence; *‘I would say that my health currently is’,* on a five-point scale from poor to good. This item was adapted [20] and has been used reliably in physical activity research [21].

### Knowledge of MVPA guidelines

Participants were asked; *‘What are the physical activity guidelines?’* An open text box enabled participants to respond with any information they felt was appropriate. Individuals who stated 150 minutes/week were labelled as having accurate knowledge regardless of whether or not an appropriate description of intensity was included.

### Awareness

Awareness of personal MVPA behaviour was calculated by first asking participants; *‘In the past week, on how many days have you done a total of 30 min or more of physical activity, which was enough to raise your breathing rate? This may include sport, exercise and brisk walking or cycling for recreation or to get to and from places, but should not include housework or physical activity that may be part of your job’*. This item has been validated by Milton et al. [22]. Response options from one to seven were provided. Next, participants responded to the statement; *‘My level of physical activity is…’* on a five-point scale from very low to very high [16]. Individuals were labelled as being accurate if their responses to these two items matched i.e. they reported engaging in MVPA on less than five days and described their activity as being less than sufficient (3 on the Likert scale). Individuals were labelled as inaccurate if their responses to the two items did not match i.e. they engaged in MVPA on less than five days but described their activity as being sufficient or better. Respondents could not see the awareness and knowledge questions at the same time.

### Subjective norms

The stem *‘Most of the people who are important to me….’* was followed by the following three items *‘think I should engage in more MVPA’; ‘engage in sufficient MVPA themselves’; ‘influence my decisions regarding MVPA’.* These questions were answered on a 7-point scale from strongly agree to strongly disagree and were selected based on guidance [23].

### Intensity of walking

In addition participants were asked; *‘What effort would you associate with walking?’* This was answered on a 15-point scale ranging from no exertion at all to maximal exertion [24]. Responses were divided into two categories with 1–7 labelled as below moderate and 8–15 as moderate-to-vigorous.

#### Analysis

All statistical analyses were performed using IBM SPSS Statistics Package 21.0. Descriptive statistics were performed to characterise the demographic variables and chi-square analyses for non-parametric data examined differences between adults with accurate and inaccurate awareness of personal MVPA engagement. Logistic regression models were developed to examine the hypotheses with α set at .05. Variables were dichotomised for entry into logistic regression models. The first model included awareness of personal physical activity (accurate versus inaccurate) as the dependent variable and knowledge of MVPA guidelines, subjective norms and self-rated health entered as predictor variables. In the second model the variable describing the perception of effort associated with walking was entered as a predictor of awareness. Both models controlled for all measured demographic variables. Odds ratios are reported for the multivariate models. Following the logistic regression analysis, the inaccurate awareness variable was divided into those who overestimated and those who underestimated their MVPA. Chi-squared analysis was then used to further explore results.

## Results

1,724 adults responded to all questions. These individuals did not differ demographically from those who started the survey but did not complete it. The sample was 70% female, 57% aged under 45, 93% White and 69% in full-time employment. 62% reported their health to be above average, while 62% demonstrated accurate awareness of their own physical activity level, only 18% correctly reported the MVPA guidelines and 51% reported high subjective norms towards MVPA. Table 1 reports group characteristics for adults with accurate and inaccurate awareness of their MVPA level. Results from chi-square analysis examining significant differences between groups are also reported in Table 1. Amongst adults with accurate awareness of their own MVPA engagement there was a greater percentage of adults aged under 45, married and reporting above average health, high subjective norms and a regular walking pace of moderate or vigorous relative to adults with inaccurate awareness.

**Table 1:** **Descriptive statistics stratified according to awareness of personal MVPA engagement**

**Accurate awareness (N = 1083)**		**Inaccurate awareness (N = 641)**		**Chi-square p value**
**Male**	29.8%	**Male**	32.6%	.14
**Aged under 45**	55.3%	**Aged under 45**	61.7%	.003
**White ethnicity**	92.4%	**White ethnicity**	92.7%	.34
**Married**	54.7%	**Married**	49.2%	.04
**Higher education**	93%	**Higher education**	92.7%	.47
**Full-time employment**	68.9%	**Full-time employed**	67.2%	.23
**Above average health**	55.2%	**Above average health**	72.1%	.001
**Accurate knowledge of guidelines**	18.1%	**Accurate knowledge of guidelines**	18.1%	.51
**High subjective norm**	52.7%	**High subjective norm**	47.5%	.04
**Moderate walking pace**	29.7%	**Moderate walking pace**	27%	.002

Table 2 reports results from the logistic regression models. Model 1 tested the hypothesised associations between awareness, knowledge, health status and subjective norms, controlling for demographic variables. Results revealed that adults with a high subjective norm were 84% more likely than adults with low subjective norms to have an accurate awareness of their MVPA level (OR = 1.84, CI: 1.29, 2.63, *p* = .001). Individuals who self-reported their health as being sufficient or less than sufficient were 29% more likely than those reporting good health to have accurate awareness of their MVPA level (OR = .71, CI: .53 .97, *p* = .001). Model 2 examined associations between awareness and the novel variable normal walking intensity, whilst controlling for demographic variables. The novel walking variable was included in a separate model (Model 2) because it is an entirely novel variable for which no directional hypothesis was made. Results showed that, relative to adults who reported their walking as normally being of a light intensity, adults who typically performed walking at a moderate-to-vigorous intensity were 31% more likely to have accurate awareness (OR = 1.31, CI: 1.05, 1.63, *p* = .02). In addition, chi-square analysis identified a significantly higher proportion of overestimators amongst those who reported usually walking at a light intensity relative to those who reported usually walking at a moderate-to-vigorous intensity (*p* = .01).

**Table 2:** **Logistic regression predicting accurate awareness of engagement with MVPA from independent variables (n = 1,724)**

**Predictor**	**Β**	**Standard error**	**Wald**	**Odds ratio**	**Lower 95% CI limit**	**Upper 95% CI limit**
Model 1: Hypothetical predictors of accurate awareness of engagement with MVPA						
Gender (male)	.12	.11	1.26	1.13	.91	1.41
Age (45 and over)	.16	.11	2.08	1.17	.95	1.45
Ethnicity (white)	-.09	.20	.22	.91	.62	1.34
Marital status (married)	.14	.11	1.75	1.15	.93	1.42
Education (low education i.e. highest level of education is completing secondary school)	.08	.2	.14	1.08	.73	1.61
Employment (part-time or unemployed)	-.01	.11	.00	.99	.80	1.24
Knowledge (accurate)	.06	.13	.22	1.06	.82	1.38
Subjective norms* (high)	.61	.18	11.44	1.84	1.29	2.63
Health* (above average)	-.34	.16	4.74	.71	.53	.97
Model 2: Exploratory predictor of accurate awareness of engagement with MVPA						
Age* (45 and over)	.23	.11	4.45	1.25	1.02	1.39
Gender (male)	.11	.11	1.10	1.12	.91	1.54
Ethnicity (white)	-.05	.19	.06	.96	.66	1.39
Marital status (married)	.15	.11	2.04	1.16	.95	1.43
Education (low education i.e. highest level of education is completing secondary school)	.05	.2	.07	1.05	.71	1.56
Employment (part-time or unemployed)	-.02	.11	.04	.98	.79	1.21
Walking intensity* (moderate or vigorous)	.27	.11	5.89	1.31	1.05	1.63

Reference group is given in brackets. *indicates a significant difference at the p < .05 level.

## Discussion

Many mass media physical activity campaigns message physical activity guidelines in a bid to motivate more adults to engage in 150 minutes a week of MVPA. For instance, the marketing strategy of the UK’s leading national health campaign Change4Life, states that; *“before we can expect behaviour change on any significant scale, people will need to know what they need to do to change”* [25]. Thus, for inactive individuals to be motivated to engage in more MVPA they may need to know how much is recommended and how their behaviour deviates from this. The present research found that accurate knowledge of MVPA guidelines was not related to adults’ awareness of their own physical activity behaviour. This is a pertinent finding as it suggests that factors other than knowledge of the amount and intensity of physical activity recommended for health may be more important when informing accurate perceptions around whether individuals are themselves sufficiently active. It supports previous suggestions that individuals with misperceptions about their physical activity behaviour do not pay attention to health messages about physical activity because they do not believe these messages to be relevant to them [26]. Knowledge of physical activity guidelines within this sample of UK adults was generally low (only 18% knew MVPA guidelines) and previously reported comparisons with 2011 data suggested that little improvement has been made over three years of focused promotional efforts [27]. The provision of information on physical activity recommendations appears to fail to improve both adults’ knowledge of guidelines and their awareness of their current engagement with physical activity. Messaging strategies which focus on creating knowledge of physical activity guidelines to motivate increased engagement in MVPA are therefore unlikely to be effective.

The present study did find that subjective norms which favoured MVPA were associated with more accurate perceptions of MVPA engagement. These subjective norms may be shaped through adult’s perceptions that their significant others (friends/family) believe they should engage in more MVPA or through having significant others who are themselves active. Previous findings found that individuals who misperceived their physical activity level used social referents who were inactive or low active [16]. Active peers may improve awareness by providing a tangible means of comparing ones behaviour to the ideal. While government sponsored information on guidelines given through campaigns may be dismissed by the average UK adult as not being personally applicable, the sight of an active friend or compulsion from a family member to engage in MVPA may alternatively be relatable and informative. Indeed, the three-year social marketing plan for Change4Life states; ‘we are more likely to adopt a habit or behaviours if we feel that it is how “people like me” normally behave’ [28]. Future campaign strategies may be more successful by highlighting how individuals have achieved physical activity guidelines using examples from within society and individual communities. Such a benchmark was included in a 2009 marketing strategy for Change4Life - “use mass media to bring the results to life and to tell people where they and their neighbours stand in relation to the nation” [25]; however, this focus has yet to emerge in the main campaign adverts to date.

Previous studies have found a normal weight status to be associated with the overestimation of physical activity level [11,29]. The present study investigated health status more generally and found that adults who perceive themselves to be healthy tend to overestimate their engagement in physical activity. Vähäsarja et al. [30] found subjective markers of health such as weight status and fitness had a stronger association with adults’ perceived need to increase physical activity than objective health markers, with those who believed themselves to be overweight or unfit being more likely to believe that they engaged in insufficient physical activity and that they needed to engage in more physical activity. Adults’ perceptions of their own health therefore seem important as a motivational driver for physical activity. Future campaigns may need to educate adults on the importance of physical activity for all and not just for those who are overweight or in poor health.

Finally, adults whose usual walking pace was described as being of at least a moderate intensity were more likely to accurately perceive their level of engagement with MVPA. Many MVPA campaigns provide walking as an exemplar despite evidence that walking is usually performed at a light intensity [17-19]. The present study found that adults who reported their usual walking pace to be light were more likely to overestimate their engagement in MVPA. Adults who typically walk slowly may erroneously believe they meet MVPA guidelines, especially if they believe that all walking is guideline-fulfilling. Campaigns such as Change4Life which promote messages such as; *“Swap four wheels for my own two feet when I go to the shops… to get me going for 150 minutes a week [advert 2011]”,* may contribute to misperceptions among ‘slow walkers’ and resultantly fail to motivate improved behaviour. This is problematic as many campaigns with the goal of promoting MVPA foreground walking in their messages but, as is apparent in the above message, fail to mention intensity. While an increase in walking, even at a slow pace, would represent a positive behavioural shift [31], campaigns which do promote walking as MVPA specifically should emphasise the intensity that is required for walking to qualify as health-enhancing MVPA and avoid general or vague messages to promote walking.

The present study reports cross-sectional data so conclusions regarding causality cannot be made. Adults’ awareness of their MVPA level was calculated using self-reported engagement with MVPA. Thus, inaccurate reporting of MVPA may also contribute to adults inaccurate perceptions of their MVPA engagement. Future studies should use objective measures of MVPA. Further, the measure of knowledge of guidelines was intentionally vague in order to capture unprompted knowledge. It is possible that some participants failed to provide the response of 150 minutes a week because they recalled physical activity guidelines other than those for health (e.g. guidelines for colon cancer). The proportion of individuals knowing such guidelines is likely to be small, thus the influence of this on our findings are likely to be minimal. Further, the number of subscribers to the list likely overestimates the sampling frame as the number of valid email addresses is unknown, junk/spam filters divert emails and it is possible that the same people have subscribed to different lists. While the response rate is therefore likely underestimated, the data collection method precludes collection of meaningful non-response data. The measure of physical activity behaviour was self-reported and this may have introduced measurement error into our calculation of physical activity awareness. The weakness of this measure could therefore have influenced our findings. On the other hand, the sample size was large and demographically representative of the UK population (based on comparisons with freely available 2008 Health Survey for England data). This is the first research to explore associations between prominent physical activity campaign messages, knowledge of physical activity guidelines, subjective norms and adults’ awareness of their MVPA engagement. Further study could explore different methods of questionnaire delivery e.g. pen and paper. The motivational implications of the findings and behavioural outcomes should also be further explored.

## Conclusions

We found high subjective norms and below average health status to be associated with adult’s perceptions of their own MVPA engagement. Unexpectedly, knowledge of MVPA guidelines was not associated with accurate awareness of engagement in MVPA. This suggests that the promotion of MVPA, through campaign messages centred on MVPA health guidelines, may not be an effective strategy for improving adult’s knowledge of recommended MVPA or awareness of their own engagement in MVPA. This could negatively influence motivation and further involvement in MVPA. These findings have substantial applied relevance for future MVPA campaigns. While the ultimate aim is for adults to achieve 150 minutes a week of MVPA this goal is a long way from being reached and simple transmission of this goal to the public via campaign messages is unlikely to improve behaviour. Cohesion is needed between academics, campaign developers and health promoters to develop evidence-based messaging strategies which effectively promote increased MVPA and surreptitiously move adults towards meeting recommendations.
